# Amorphigenin from *Amorpha fruticosa* L. Root Extract Induces Autophagy-Mediated Melanosome Degradation in mTOR-Independent- and AMPK-Dependent Manner

**DOI:** 10.3390/cimb44070196

**Published:** 2022-06-29

**Authors:** Ki Won Lee, Dang Thi Nguyen, Minju Kim, Si Hyeon Lee, Seyeon Lim, Jisu Kim, Ki Hun Park, Jeong Yoon Kim, Jiyun Yoo, Cheol Hwangbo, Kwang Dong Kim

**Affiliations:** 1 PMBBRC, Gyeongsang National University, Jinju 52828, Korea; leemaskup@naver.com; 2 Division of Applied Life Science, Gyeongsang National University, Jinju 52828, Korea; dthinguyen96.cnsh@gmail.com (D.T.N.); kmj941226@naver.com (M.K.); 2tlgus@naver.com (S.H.L.); tpdus1643@naver.com (S.L.); qmffor2722@naver.com (J.K.); khpark@gnu.ac.kr (K.H.P.); yooj@gnu.ac.kr (J.Y.); chwangbo@gnu.ac.kr (C.H.); 3 Department of Pharmaceutical Engineering, Gyeongsang National University, Jinju 52725, Korea; jykim21@gnu.ac.kr; 4 Zeeum Co., Ltd., 17-17, 208, Chemdangwagi-ro, Buk-gu, Gwangju 61011, Korea

**Keywords:** *Amorpha fruticosa* L., depigmentation, autophagy, amorphigenin, AMPK

## Abstract

In this study, we investigated the depigmentation effect of *Amorpha fruticosa* L. root extract (RE), an herbal medicine. *A. fruticosa* RE significantly induced depigmentation in α-MSH-treated B16F10 cells at noncytotoxic concentrations. Further, the RE decreased the protein levels of the melanosomal proteins Tyr and Pmel without decreasing their transcript levels. We found that MG132, a proteasome complex inhibitor, was unable to rescue the protein levels, but PepA/E-64D (a lysosomal enzyme inhibitor), 3-MA (a representative autophagy inhibitor), and *ATG5* knockdown effectively rescued the protein levels and inhibited the depigmentation effect following RE treatment. Among rotenoids, amorphigenin composed in the RE was identified as a functional chemical that could induce depigmentation; whereas rapamycin, an mTOR inhibitor and a nonselective autophagy inducer, could not induce depigmentation, and amorphigenin effectively induced depigmentation through the degradation of melanosomal proteins. Amorphigenin activated AMPK without affecting mTOR, and knockdown of AMPK offset the whitening effect through degradation of melanosome proteins by amorphigenin. Results from this study suggested that amorphigenin can induce degradation of the melanosome through an AMPK-dependent autophagy process, and has the potential to be used as a depigmentation agent for the treatment of hyperpigmentation.

## 1. Introduction

Melanin is a pigmented biopolymer distributed in the hair, skin, and eyes. The balance between melanin synthesis and melanin degradation is thought to be important in the homeostasis of skin color. The process of melanogenesis, a melanin synthesis process that occurs in melanocytes, has been relatively well studied. The α-melanocyte-stimulating hormone (α-MSH) initiates melanogenesis by binding to the melanocortin 1 receptor (MC1R) on melanocytes [1]. Then, α-MSH induces MITF expression via activation of the cyclic adenosine monophosphate response element-binding protein (CREB) [2]. MITF regulates the expression of melanogenesis-related genes, including *tyrosinase* (*Tyr*), *tyrosinase-related protein-1* (*TRP-1*), *premelanosome* (*Pmel*), and *melan-A* [3,4,5]. The formation of the melanosome, where melanogenesis occurs, is induced through MITF-mediated gene expression, such as *Pmel*, and biogenesis of lysosome-related organelles complex *(BLOC)-1*, *BLOC-2*, *BLOC-3*, adaptor protein (*AP*)-1, and *AP-3* [6,7,8,9,10]. Whereas melanosomes surrounding the nucleus play a role in protecting against ultraviolet (UV)-mediated DNA damage [11], uncontrolled excessive melanin can cause various hyperpigmentation conditions such as melisma, sun spots, and aging spots [12,13,14]. Although melanogenesis plays an important role in determining skin color, the degradation process of already-formed melanin may also be important.

Recent work has reported that autophagy is related to skin color [15]. Autophagy is a destruction mechanism that naturally degrades unnecessary or nonfunctional cellular components under cellular physiological stress conditions [16]. Autophagy is classified as either nonselective autophagy or selective autophagy, depending on the specificity of target molecules or organelles (cargo). Several studies have reported on the selective autophagy of several organelles, including the endoplasmic reticulum, lysosome, mitochondria, nucleus, and peroxisome. In both human [17,18] and rodent [19], melanomas are often characterized by the accumulation of autophagosomes that contain preformed melanosomes (Bomirski et al. 1987), perhaps suggesting a role for autophagosomes in melanosomal degradation. Although the molecular mechanisms underlying melanosome autophagy remain unclear, several chemicals have been proposed that could induce skin depigmentation through autophagy [20,21,22,23,24].

In the present study, we investigated the effects of the root extract (RE) of *Amorpha fruticosa* L. on autophagy-mediated melanosome degradation in B16F10 cells, and determined that an active ingredient, amorphigenin, can induce depigmentation by inducing autophagy in B16F10 cells.

## 2. Materials and Methods

### 2.1. Cell Culture and Chemicals

We purchased the mouse melanoma cell line B16F10 from the American Type of Culture Collection (ATCC, Manassas, VA, USA); α-MSH, kojic acid, and MG132 from Sigma-Aldrich (St. Louis, MO, USA); trypsin–ethylenediaminetetraacetic acid (EDTA) from Lonza (Walersville, MD, USA); and 3-(4,5-dimethylthiazol-2-yl)-2,5-diphenyl tetrazolium bromide (MTT) from Amresco (Solon, OH, USA). B16F10 cells were cultured in a 5% CO_2_ humidified incubator at 37 °C in Dulbecco’s Modified Eagle Medium (DMEM; Sigma-Aldrich) supplemented with 10% fetal bovine serum (FBS; ATCC) and 1% penicillin/streptomycin (Sigma-Aldrich).

### 2.2. Cell Viability Assay

B16F10 cells were seeded in 96-well plates and incubated overnight. Following incubation, the cells were treated with the indicated concentrations of *A. fruticosa* RE for 48 h. Next, MTT was added, and the cells were incubated for another 3 h at 37 °C in a 5% CO_2_ humidified incubator. After incubation, the cell culture medium was discarded, and dimethyl sulfoxide (DMSO; Sigma-Aldrich) was added for 20 min at room temperature (RT). Cell viability was measured using a microplate reader (Bio-Rad Laboratories, Hercules, CA, USA) at a wavelength of 595 nm.

### 2.3. Melanin Assay

B16F10 cells were seeded in 6-well plates and incubated overnight. Following incubation, the cells were treated with α-MSH in the presence or absence of *A. fruticosa* RE for 48 h, harvested by trypsinization, and dissolved in 1 N NaOH containing 10% DMSO at 65 °C for 24 h. The melanin content of cells was measured using a microplate reader (Bio-Rad Laboratories) at a wavelength of 415 nm. 

### 2.4. L-DOPA Oxidation

B16F10 cells were seeded in 6-well plates and incubated overnight. Following incubation, the cells were treated with α-MSH in the presence or absence of *A. fruticosa* RE for 48 h, harvested by trypsinization, and lysed with 1% Triton X-100 in phosphate-buffered saline (PBS) for 1 h. A total of 40 μg of proteins in solution were incubated with 2 mg/mL of L-3,4-dihydroxyphenylalanine (L-DOPA) (Sigma-Aldrich) at 37 °C for 4 h. Following incubation, absorbance was measured on a microplate reader (Bio-Rad Laboratories) at a wavelength of 490 nm.

### 2.5. Preparation of Root Bark Extract and Isolation of Amorphigenin

Root bark extract (RE) was prepared by adding 20 mL ethanol per 1 g dried and chopped root bark of *A. fruticosa* and then sonicating the sample at RT for 4 h. Metabolite analysis of the RE was performed using the Vion UPLC-ESI-Q-TOF/MS system (Waters, Milford, MA, USA). For purification of amorphigenin from RE, the dried root bark of *A. fruticose* (0.5 kg) was extracted with ethanol (10 L) for one week at RT. The solution was then evaporated to obtain the concentrate (75 g). The concentrate was dissolved in water and portioned with n-hexane, ethyl acetate, and butanol in order to achieve polarity. Among the solvent fractions, the ethyl acetate (8 g) was fractionated on a silica-gel column (10 × 40 cm, 230~400 mesh, 50 g) using the n-hexane-ethyl acetate mixture to yield 8 fractions (A-H). Fraction D (1.8 g) was subjected to a reversed silica gel column (250 × 30 mm, S-5 μm, 12 nm, YMC) under prep-HPLC (Forte-R, YMC CO., LTD., Kyoto, Japan) and eluted with an isocratic of 80% MeOH at a rate of 10 mL/min to give 10 subfractions (Fr. D1-D10). Subfraction D5 (280 mg), which was enriched with amorphigenin, was further purified by recycling LC (LC 9110 NEXT, JAI Co., Ltd., Tokyo, Japan) on reversed silica-gel (250 × 30 mm, S-5 μm, 12 nm, Thermo Fisher Scientific) with a gradient condition of 0–100% acetonitrile to obtain amorphigenin as a single compound (160 mg). The amorphigenin was characterized and identified using spectroscopic methods, in addition to comparisons with published data [25].

Amorphigenin: Needles; EIMS *m*/*z* 410 [M]^+^; HREIMS *m*/*z* 410.1368 (calcd. for C_23_H_22_O_7_ 410.1336); ^1^H NMR (500 MHz, CDCl3) d 7.77 (1H, d, J = 8.55 Hz, H-11), 6.84 (1H, s, H-1), 6.44 (1H, d, J = 8.55 Hz, H-10), 6.38 (1H, s, H-4), 5.32 (1H, t, J = 9.05, H-50), 5.20 (2H, d, J = 12.9 Hz, H-8′), 4.86 (1H, m, H-6a), 4.54 (1H, m, H-6b), 4.19 (2H, d, J = 4.05 Hz, H-7′), 4.11 (1H, m, H-6a), 3.78 (1H, d, J = 4.05 Hz, H-12a), 3.69 (3H, s, OMe), 3.74 (3H, s, OMe), 3.32 (1H, m, H-4′a), 3.02 (1H, m, H-4′b); ^13^C NMR (125 MHz, CDCl3) d 189.3 (C-12), 167.3 (C-9), 158.3 (C-9), 149.9 (C-2), 147.1 (C6′), 144.3 (C-3), 130.40 (C-11), 113.9 (C-11a), 113.3 (C-8), 112.9 (C-8′), 110.8 (C1), 105.32 (C-10), 105.3 (C-12b), 101.3 (C-2), 85.9 (C-5′), 72.6 (C-6a), 66.6 (C-6), 63.2 (C-7′), 45.12 (C-12a), 29.7 (C-4′). 

### 2.6. RNA Extraction and Polymerase Chain Reaction (PCR)

After chemical treatment of the B16F10 cells, the total RNA was extracted using an RNeasy mini kit (Qiagen, Hilden, Germany). Following RNA extraction, we synthesized complementary DNA (cDNA) from 2 μg of RNA using a RevertAid First Strand cDNA synthesis kit (Thermo Fisher Scientific, Waltham, MA, USA). PCR was performed using the Solg™ e-Taq DNA Polymerase kit (SolGent Co. Ltd., Daejeon, Korea). The following primers were used: *MITF* forward primer, 5′-TACAGTCACTACCAGGTGCAG-3′; *MITF* reverse primer, 5′-CCATCAAGCCCAAAATTTCTT-3′, *Tyr* forward primer, 5′-GGCCAGCTTTCAGGCAGAGGT-3′; *Tyr* reverse primer, 5′-TGGTGCTTCATGGGCAAAATC-3′; *Trp-1* forward primer, 5′-GCTGCAGGAGCCTTCTTTCTC-3′; *Trp-1* reverse primer, 5′-AAGACGCTGCACTGCTGGTCT-3′; *Glyceraldehyde 3-phosphate dehydrogenase* (*Gapdh*) forward primer, 5′-CCATCACCATCTTCCAGGAG-3′; *Gapdh* reverse primer, 5′-ACAGTCTTCTGGGTGGCAGT-3′. We used a SsoFast™ Evagreen supermix (Bio-Rad Laboratories) for quantitative reverse transcription polymerase chain reaction (qRT-PCR). We analyzed qRT-PCR products using a CFX96™ real-time PCR system (Bio-Rad Laboratories). *Tyr* and *Pmel C*_T_ values were normalized against *C*_T_ values of *β-actin*. The following primers were used: *β-Actin* forward primer, 5′-GGTCATCACTATTGGCAACG-3′; *β-Actin* reverse primer, 5′-ACGGATGTCAACGTCACACT-3′; *Tyr* forward primer, 5′-GCCCAGCATCCTTCTTC-3′; *Tyr* reverse primer, 5′-TAGTGGTCCCTCAGGTGTTC-3′; *Pmel* forward primer, 5′-ACCCAACTTGTTGTTCCTGG-3′; *Pmel* reverse primer, 5′-GTGCTACCATGTGGCATTTG-3′. 

### 2.7. Western Blotting

We extracted total proteins using CETi lysis buffer (Translab, Daejeon, Korea). Following 8–15% sodium dodecyl sulfate polyacrylamide gel electrophoresis (SDS-PAGE), proteins were transferred onto a polyvinylidene fluoride (PVDF) membrane (Merck Millipore, Burlington, MA, USA). Next, the PVDF membrane was blocked with 5% skim milk in Tris-buffered saline with 0.1% Tween 20 (TBS-T) for 1 h at RT and then incubated with the following primary antibodies overnight at 4 °C: anti-MITF, anti-phospho-mTOR (Ser 2448), anti-mTOR, anti-phospho-AMPK (Thr 172), anti-AMPK antibody (Cell Signaling Technology, Beverly, MA, USA), anti-Pmel antibody (EP4863(2)) (Abcam, Cambridge, UK), anti-ATG5 and anti-α-tubulin (Sigma-Aldrich), and anti-Tyr antibody (LifeSpan BioScience, Seattle, WA, USA). Signal detection was performed via enhanced chemiluminescence (ECL; Bio-Rad Laboratories).

### 2.8. Confocal Microscopy

B16F10 cells were seeded on a coverslip and incubated overnight. Following incubation, the cells were treated with RE, fixed in 4% paraformaldehyde for 15 min at RT, and permeabilized using 0.1% Triton X-100 for 10 min at RT. Following permeabilization, we used 1% bovine serum albumin (BSA) for blocking and then incubated the cells with anti-Trp-1 primary antibody (Santa Cruz Biotechnology, CA, USA) overnight at 4°C. The cells were treated with anti-mouse–fluorescein isothiocyanate (FITC; Sigma-Aldrich) for 1 h at RT and then treated with 20 μg/mL of 4′,6-diamidino-2-phenylindole (DAPI; Sigma-Aldrich) for 10 min at RT. The cells were mounted on a glass slide with VECTASHIELD^®^ mounting media (Vector Laboratories, CA, USA), and images were acquired on an Olympus FluoView™ FV1000 confocal microscope (Olympus, Tokyo, Japan).

### 2.9. Gene Silencing

The validated small interfering RNAs (siRNAs) for mouse ATG5 (5′-ACCGGAAACUCAUGGAAUA-3′) [26], for mouse AMPKα1/α2 (5′-AUGAUGUCAUGGUGAAUUU-3′), and for GL2 luciferase (5′-CGUACGCGGAAUACUUCGA-3′) were synthesized from Genolution (Seoul, Korea). The siRNAs were transfected into B16F10 cells using lipofectamine RNAiMAX according to the manufacturer’s protocols (Invitrogen, Carlsbad, CA, USA).

## 3. Results

### 3.1. A. fruticosa RE Induces Depigmentation in α-MSH-Stimulated B16F10 Cells

**Figure 1:**
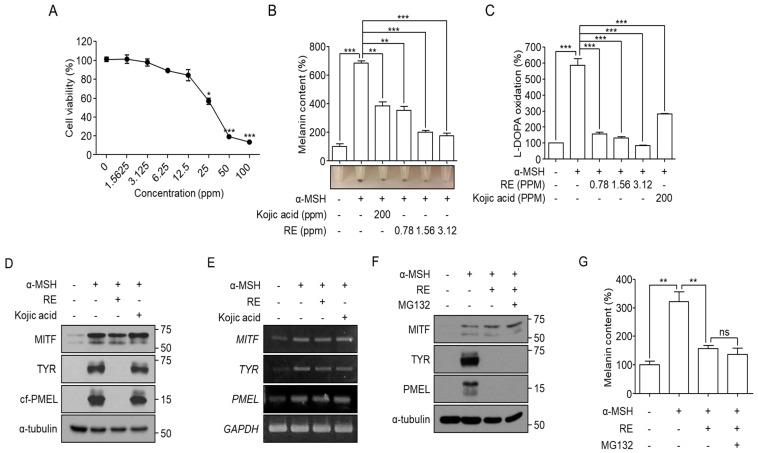
**Depigmentation effect of *A. fruticosa* L. RE in α-MSH-stimulated B16F10 cells.** (**A**) B16F10 cells were incubated with *A. fruticosa* RE for 48 h. Following incubation, cell viability was measured using an MTT assay. (**B**,**C**) B16F10 cells were treated with 1 μM α-MSH in the presence or absence of kojic acid or *A. fruticosa* RE for 48 h. After harvesting, cellular melanin content (**B**) and L-DOPA oxidation (**C**) were measured. (**D**,**E**) B16F10 cells were treated with 1 μM α-MSH in the presence or absence of 200 ppm of kojic acid as a positive control or 3.12 ppm of *A. fruticosa* RE for 48 h. The indicated protein (**D**) or mRNA expression (**E**) levels were analyzed by Western blotting or RT-PCR, respectively. (**F**,**G**) Following treatment of B16F10 cells for 48 h with 3.12 ppm of *A. fruticosa* RE in the presence or absence of 120 nM MG132, the indicated protein levels (**F**) and melanin content (**G**) were analyzed. Data were obtained from at least three independent experiments, and the values represent the mean ± SEM. ns, not significant, * *p* < 0.05, ** *p* < 0.01, and *** *p* < 0.001 according to *t*-tests.

To determine the cytotoxicity of *A. fruticosa* RE in B16F10 cells, we treated B16F10 cells with various concentrations of *A. fruticosa* RE for 48 h and then measured cell viability using an MTT assay. We found that *A. fruticosa* RE-mediated cytotoxicity was significantly increased from 12.5 ppm (Figure 1A). To determine whether *A. fruticosa* RE affects melanogenesis, B16F10 cells were stimulated with α-MSH in the presence or absence of the indicated concentrations of *A. fruticosa* RE for 48 h. Kojic acid (200 ppm), a Tyr inhibitor, was used as a positive control for depigmentation. *A. fruticosa* RE effectively inhibited α-MSH-mediated accumulation of melanin and cellular L-DOPA oxidation (Figure 1B,C). This result indicated that *A. fruticosa* RE could inhibit melanogenesis through a decrease in intracellular tyrosinase activity. We found that α-MSH upregulated MITF, Tyr, and Pmel expression, while *A. fruticosa* RE effectively decreased levels of Tyr and Pmel, but not MITF, proteins (Figure 1D). *A. fruticosa* RE did not affect expression at the messenger RNA level for each gene (Figure 1E). This observation suggested that *A. fruticosa* RE-mediated depigmentation may be due to the degradation of melanosomal proteins involved in melanin synthesis. We inhibited the ubiquitin-proteasome system (UPS) that induces cellular protein degradation by treating α-MSH-treated B16F10 cells with a proteasome inhibitor, MG132, along with *A. fruticosa* RE. We found that MG132 failed to rescue the reduction in melanosomal protein levels induced by *A. fruticosa* RE (Figure 1F), and did not inhibit depigmentation induced by *A. fruticosa* RE treatment (Figure 1G). This result indicated that *A. fruticosa* RE inhibits melanogenesis through a mechanism in which melanosome-related proteins are degraded in a UPS-independent manner.

### 3.2. A. fruticosa RE-Mediated Depigmentation Depends on Autophagy

**Figure 2:**
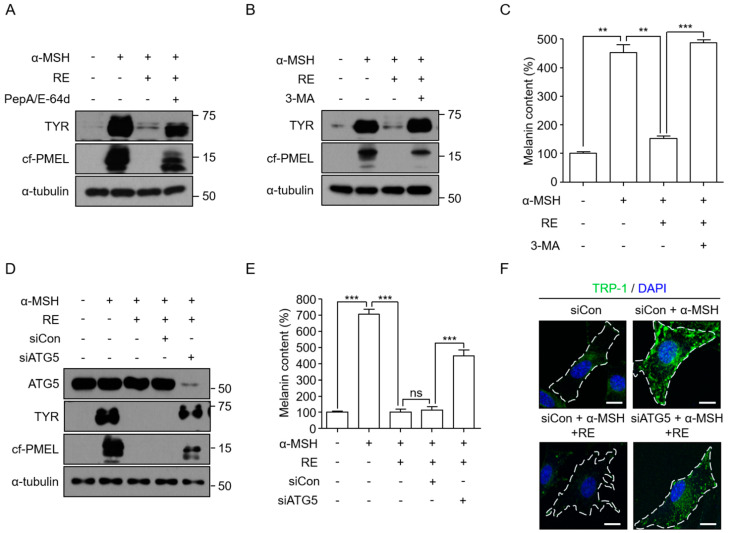
**Depigmentation effect of*A. fruticosa*RE depends on autophagy-mediated melanosome degradation.** (**A**) B16F10 cells were treated with 1 μM α-MSH in the presence or absence of 3.12 ppm of *A.* fruticosa RE for 24 h. Cells were further treated with PepA/E-64d (10 μg/mL each) for an additional 24 h. The indicated protein expression levels were analyzed using Western blotting. (**B**,**C**) Following treatment of B16F10 cells for 48 h with 3.12 ppm of *A. fruticosa* RE in the presence or absence of 800 µM 3-MA, the indicated protein levels (**B**) and melanin content (**C**) were analyzed. (**D**,**E**) B16F10 cells were transfected with siRNA for 24 h and then treated with 1 μM α-MSH in the presence or absence of 3.12 ppm of *A.* fruticosa RE for 48 h. Subsequently, analyses of protein expression level (**D**), melanin content (**E**), and confocal microscopy images (**F**) were performed. Data were obtained from at least three independent experiments, and the values represent the mean ± SEM. Note: ns, not significant; ** *p* < 0.01 and *** *p* < 0.001 according to *t*-tests. Scale bar = 10 μm.

To determine whether lysosome-dependent proteolysis is involved in *A. fruticosa* RE-mediated depigmentation, B16F10 cells were stimulated with α-MSH in the presence of *A. fruticosa* RE and pepstatin A1 (PepA)/E-64d, an inhibitor of lysosomal proteinase. The degradation of melanosomal proteins by *A. fruticosa* RE was inhibited by PepA/E-64d (Figure 2A). We also investigated whether the autophagy pathway was involved in the *A. fruticosa* RE-mediated depigmentation by inhibiting autophagy in B16F10 cells through treatment with 3-MA or ATG5 knockdown. The autophagy inhibitor 3-MA not only restored the level of melanosomal proteins in the *A. fruticosa* RE-treated B16F10 cells, but also counteracted the *A. fruticosa* RE-mediated depigmentation effect (Figure 2B,C). Knockdown of the expression of the *ATG5* gene, an essential gene for autophagy [27], also inhibited the degradation of melanosomal proteins and depigmentation by *A. fruticosa* RE (Figure 2D,E). The Trp-1-associated dotty subcellular pattern, representing melanosomes, decreased significantly following *A. fruticosa* RE treatment, while *ATG5* knockdown rescued the decrease in melanosomes (Figure 2F). Together, these results suggested that an autophagy process was involved in *A. fruticosa* RE-mediated degradation of melanosomal proteins and depigmentation in the α-MSH-stimulated B16F10 cells.

### 3.3. Identification of Amorphigenin as a Functional Metabolite in A. fruticosa RE

**Figure 3:**
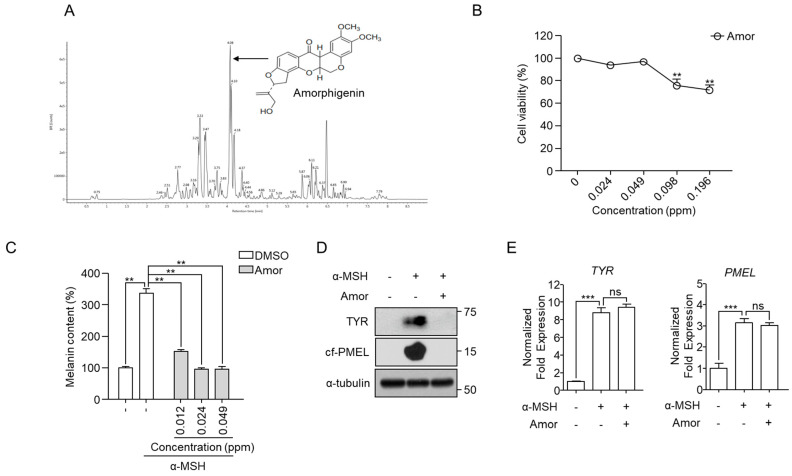
**Amorphigenin as a functional metabolite in*A. fruticosa*L.RE-mediated depigmentation.** (**A**) Chromatographic analysis of *A. fruticosa* RE using Vion UPLC-ESI-Q-TOF/MS system. (**B**) B16F10 cells were treated with various concentrations of amorphigenin (Amor) for 48 h, and cell viability was measured using an MTT assay. (**C**,**E**) B16F10 cells were treated with 1 μM α-MSH in the presence or absence of the indicated concentrations of Amor for 48 h, and the melanin content (**C**), the indicated protein expression levels (**D**), and mRNA expression levels (**E**) were measured. Data were obtained from at least three independent experiments, and the values represent the mean ± SEM. Note: ns, not significant; ** *p* < 0.01 and *** *p* < 0.001 according to *t*-tests.

A previous study [25] identified several flavones and rotenoids in *A. fructicosa* roots. Amorphigenin was a major rotenoid (Figure 3A) and showed a depigmentation effect in B16F10 cells. Amorphigenin showed significant cytotoxicity above 0.098 ppm in B16F10 cells. For this reason, we selected 0.049 ppm as the maximum concentration in our experiments (Figure 3B). Amorphigenin effectively induced depigmentation (Figure 3C) and decreased Tyr and Pmel levels (Figure 3D) without affecting mRNA (Figure 3E) levels, as observed for *A. fruticosa* RE. These result suggested that amorphigenin is a functional metabolite that can induce depigmentation in *A. fruticosa* RE through degradation of melanosomal proteins.

### 3.4. Amorphigenin-Mediated Depigmentation Depends on Autophagy

**Figure 4:**
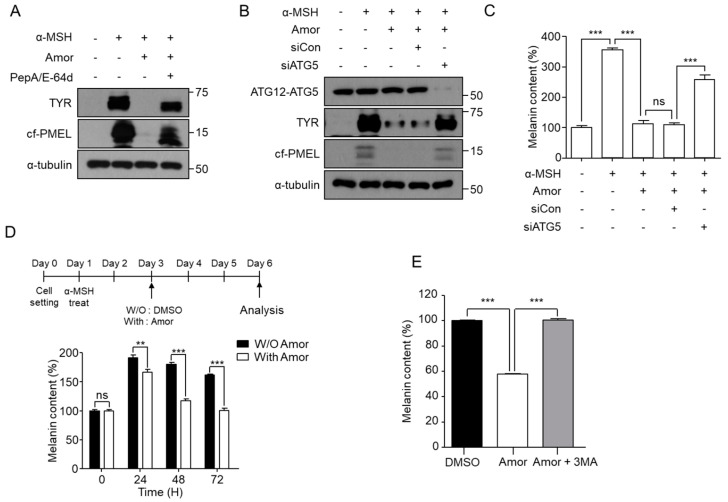
**Amorphigenin induces autophagy-mediated depigmentation.** (**A**) Following treatment with 1 μM α-MSH with 0.012 ppm of amorphigenin (Amor) for 24 h, the cells were further treated with PepA1/E-64d (10 μg/mL each) for an additional 24 h. Next, the indicated protein levels were analyzed using Western blotting. (**B**,**C**) siRNA was transfected into B16F10 cells, and after 24 h, the transfected cells were treated with 1 μM α-MSH in the presence or absence of 0.012 ppm amorphigenin for 48 h. Then, the indicated protein levels (**B**) and melanin content (**C**) were measured. After experiments were performed according to the schematic, the melanin content was measured (**D**,**E**). Data were obtained from at least three independent experiments, and the values represent the mean ± SEM. Note: ns, not significant; ** *p* < 0.01 and *** *p* < 0.001 according to *t*-tests.

To determine whether an autophagy process is involved in amorphigenin-mediated depigmentation, α-MSH-stimulated B16F10 cells were treated with amorphigenin in the presence or absence of PepA/E-64d. PepA/E-64d effectively inhibited the amorphigenin-induced decrease in Tyr and Pmel expression levels (Figure 4A). The amorphigenin-mediated degradation of melanosomal proteins and depigmentation effect also disappeared in ATG5-knockdown cells (Figure 4B,C). We reasoned that if amorphigenin showed a depigmentation effect via autophagy, it should show the same depigmentation effect in B16F10 cells in which melanosomes were already formed, because autophagy can degrade preformed cellular components. To test whether amorphigenin has a depigmentation effect on preformed pigments, we treated B16F10 cells with amorphigenin on day 2 following α-MSH stimulation. We found that amorphigenin induced a significant loss of the preformed melanin in a time-dependent manner (Figure 4D), and 3-MA could inhibit the depigmentation effect induced by amorphigenin (Figure 4E). These results indicated that amorphigenin is a functional chemical in *A. fruticosa* RE that can induce depigmentation through autophagy-mediated melanosome degradation in α-MSH-treated B16F10 cells.

### 3.5. Amorphigenin Induces Degradation of Melanosomes in an mTOR-Independent and AMPK-Dependent Manner

**Figure 5:**
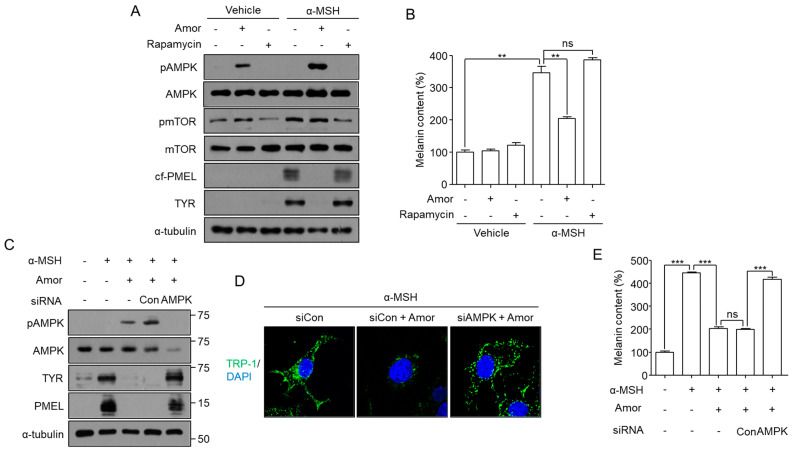
**AMPK-dependent melanosome autophagy induced by amorphigenin.** (**A**,**B**) B16F10 cells were treated with 0.012 ppm of amorphigenin (Amor) or 0.5 µM of rapamycin in the presence or absence of 1 µM of α-MSH for 48 h, and the indicated protein expression levels (**A**) and melanin content (**B**) were analyzed. To inhibit AMPK activity, validated AMPK siRNA, as described in the Materials and Methods section, was transfected into B16F10 cells, and then the cells were treated with amorphigenin for 48 h after α-MSH stimulation (**C**–**E**). To analyze melanosome degradation, TRP-1 spot formation was analyzed in the indicated experimental groups (**D**). Data were obtained from at least three independent experiments, and the values represent the mean ± SEM. Note: ns, not significant; *** *p* < 0.005 and ** *p* < 0.01 according to *t*-tests. Scale bar = 10 μm.

To test whether autophagy-mediated depigmentation by amorphigenin was dependent on the mTOR/AMPK pathway, the phosphorylations of mammalian target of rapamycin (pmTOR) and AMP-activated protein kinase (AMPK) were analyzed following amorphigenin or rapamycin treatment. Whereas rapamycin induced mTOR inhibition and but depigmentation, amorphigenin induced AMPK activation and depigmentation but not mTOR inhibition (Figure 5A,B). To confirm whether amorphigenin-induced AMPK activation was essential for depigmentation, levels of melanosomal proteins and melanin content were analyzed in B16F10 cells transfected with siAMPK. Amorphigenin-mediated downregulation of the melanosomal proteins Tyr and cleaved PMEL was effectively rescued following AMPK knockdown (Figure 5C), and AMPK-dependent melanosome degradation was induced by amorphigenin, as confirmed by confocal microscopy analysis (Figure 5D). The depigmentation effect of amorphigenin was also inhibited by AMPK knockdown (Figure 5E). 

## 4. Discussion

*A. fruticosa* roots contain various polyphenols, such as rotenoids, prenylated flavanones, isoflavones, and stilbenes [28], and may play a role in several biological properties, including antitumor [29], anti-inflammatory [30], and antidiabetic activities [31]. In this study, we investigated autophagy-mediated depigmentation by *A. fruticosa* RE in B16F10 cells, and identified amorphigenin as a functional compound. 

Melanosomes are specific organelles in which melanogenesis occurs in melanocytes [32]. Following melanogenesis, melanosomes are transferred from melanocytes to keratinocytes [33]. Skin color is determined by the melanin content of melanosomes, so targeting melanosomes is important for skin-whitening applications [34,35]. Many skin depigmentation agents have been identified on the basis of tyrosinase inhibition. *A. fruticosa* RE also inhibits L-DOPA oxidation, which correlates with a decrease in tyrosinase activity [36]. The *A. fruticosa* RE-induced decrease in intracellular melanin content may be due to the direct inhibition of Tyr activity [37,38,39], inhibition of Tyr modification [40,41], or a decrease in Tyr expression [42,43]. The decrease in melanosomal protein levels by RE was due to a decrease in protein stability and not a decrease in their transcript levels. Generally, the stability of intracellular proteins is mainly regulated through the UPS [44]. The *A. fruticosa* RE-mediated decrease in melanosomal proteins levels might be due to the UPS, because *A. fruticosa* RE cannot decrease the mRNA levels for the proteins. However, MG132, a representative cell-permeable proteasome inhibitor that works against the UPS [45], could not rescue *A. fruticosa* RE-mediated protein decrease. Another protein degradation process is the lysosome-dependent mechanism [46]. Autophagy in particular is a type of homeostatic mechanism that degrades cellular proteins or cellular organelles in a lysosome-dependent manner [47]. Autophagy can capture cytosolic contents in double-membrane vesicles called autophagosomes. ATG5 forms a protein complex with ATG12 and ATG16L during autophagosome membrane elongation [27]. In the final step of autophagy, autophagosomes can fuse with lysosomes to form autolysosomes, which in turn degrade the captured cytosolic contents through lysosomal protease. Inhibition of both autophagosome formation through *ATG5* knockdown or autophagy-mediated degradation using lysosomal protease inhibitors can block the degradation of Tyr and Pmel induced by *A. fruticosa* RE or its component, amorphignin. These results suggest that amorphigenin induces the degradation of melanosomes through an autophagy process. Interconnection among mTOR, AMPK, and unc-51-like 1/2 (ULK1/2) play an essential role in macroautophagy [48]. Rapamycin, a representative nonselective autophagy inducer and mTOR inhibitor, did not induce depigmentation in α-MSH-stimulated B16F10 cells. Amorphigenin-mediated melanosome degradation was effectively inhibited by AMPK knockdown, regardless of mTOR. AMPK has been reported to play an important role in selective autophagy. AMPK deficiency results in defective mitophagy [49], and AMPK-mediated ULK1 activation is essential for the initiation of mitophagy under hypoxia conditions [50]. AMPK is localized to the mitochondrial outer membrane, and the inhibition of the AMPK activity attenuates mitophagy in skeletal muscle [51]. 

Until now, there have been suggestions regarding the possibility of selective autophagy for melanosomes, but few reports have focused on the molecular mechanisms. This study suggested that amorphigenin-induced melanosome degradation may be an AMPK-dependent selective autophagy on melanosomes. 

## Data Availability

The data used to support the findings of this study are included in the article.
